# Reported exposure to news portrayals about mental health problems and their impact: Findings from the 2025 National Survey of Stigma and Discrimination

**DOI:** 10.1177/00048674261462377

**Published:** 2026-07-10

**Authors:** Anna M Ross, Amy J Morgan, Gayle McNaught, Rachel Green, Nicola J Reavley

**Affiliations:** 1 Centre for Mental Health and Community Wellbeing, Melbourne School of Population and Global Health, The University of Melbourne, Melbourne, VIC, Australia; 2 SANE Australia, Carlton, VIC, Australia

**Keywords:** Stigma, attitudes, media, news, mental illness, mental health

## Abstract

**Objective:**

Media portrayals of people with mental illness have the power to mitigate or perpetuate stigma related to mental health. This study aimed to investigate reported real-world exposure to news media portrayals about people with mental health problems in the past 12 months and the impact of these.

**Methods:**

Data were from a nationally representative survey of 6032 Australians exploring attitudes towards people with mental health problems. Participants were asked about their exposure to positive news stories about a person with a mental health problem, as well as negative portrayals, in which someone was harmed by a person with a mental health problem. Further questions covered the sources (traditional or social media) and impact of these exposures.

**Results:**

Regression models were used to explore sociodemographic predictors of impact. Most participants reported exposure to negative news portrayals (68.4%, 95% confidence interval = [66.9, 69.8]), while fewer reported exposure to positive news stories (33.7%, 95% confidence interval = [32.3, 35.2]). Most people exposed to the negative news stories reported a negative impact (69.0%, 95% confidence interval = [67.2, 70.7]), and most exposed to positive news stories reported a positive impact (80.2%, 95% confidence interval = [77.6, 82.5]). Age and gender were associated with reported impact but not lived experience of mental illness.

**Conclusions:**

Exposure to negative news stories about mental health problems was prevalent. Given their impact on news audiences broadly, negative news stories need to be accurate and responsible to mitigate negative impacts. A renewed focus on generating and promoting positive and stigma-challenging news stories is needed to increase subsequent positive impacts.

## Introduction

### The role of news media in perpetuating and mitigating stigma

Media portrayals of people with mental illness are known to influence public attitudes related to mental health problems. These portrayals can act to mitigate or perpetuate stigma towards people with mental illness, depending on their framing, tone and content. Positively framed news portrayals that aim to increase understanding of mental illness and actively challenge stigma have been found to positively influence public attitudes (Corrigan et al., 2013; Ross et al., 2019). These types of portrayals include stories highlighting the recovery journey and achievements of people with lived experience, interviews with mental health professionals to explore topics in-depth as well as stories that actively challenge stigma by addressing misconceptions in the mainstream media.

The media is also a widely recognised driver of stigma. This has been examined using rigorous experimental research designs, with controlled exposures to news stories about people with mental illness and the subsequent completion of stigma scales (e.g. Cain et al., 2014; Gwarjanski and Parrott, 2017). Negatively framed portrayals include those that link mental illness and violence, and perpetuate inaccurate stereotypes about people with mental illness, including that they are dangerous or cannot recover (Ross et al., 2019). These negative portrayals have been found to increase stigmatising attitudes, including those relating to dangerousness and unpredictability and increased belief in stereotypes about violence (Angermeyer et al., 2005; Dietrich et al., 2006; Gwarjanski and Parrott, 2017; McGinty et al., 2018). Holding these negative stereotypes about people with mental illness can also contribute to discriminatory behaviours, including social exclusion, and structural discrimination in employment, housing and healthcare (Morgan et al., 2012; Switaj et al., 2009), as well as reduce help-seeking related to mental health (Morgan and Jorm, 2009).

The impacts of media portrayals are potentially stronger on attitudes towards people with lower prevalence conditions that are more highly stigmatised, such as schizophrenia and psychosis (hereafter referred to as complex mental illness). The general population are less likely to have interpersonal contact with people with these conditions compared to more common illnesses such as depression and anxiety, and are therefore more likely to have attitudes more influenced by other information sources, especially the media (Reavley et al., 2016). Complex mental illness is most commonly portrayed in relation to violence and crime in the media (Cain et al., 2014). Such portrayals overrepresent the occurrence of these rare events and perpetuate inaccurate stereotypes about people with these conditions (Anderson et al., 2018; Cain et al., 2014; Swanson et al., 2015). This is despite the evidence showing that most people with mental illness are never violent (Fazel and Grann, 2006; Hodgins, 2008).

Despite efforts to tackle stigma, recent evidence from an Australian population survey shows stigma towards mental illness is actually increasing (Reavley et al., 2026a). Addressing media reporting practices has been identified as a priority action to mitigate stigma. This includes in key reports, such as the 2022 Lancet Commission on Ending Stigma and Discrimination in Mental Health (Thornicroft et al., 2022), and within Australia, the draft National Stigma and Discrimination Reduction Strategy (National Mental Health Commission, 2022), both of which involved extensive consultations with people with lived experience, carers and other mental health experts. Despite this recognition of the valuable role of media for population-level intervention, narratives featuring stories of recovery or the achievements of people with complex mental illness are often absent or overshadowed in mainstream media portrayals (Cain et al., 2014; Gwarjanski and Parrott, 2017; Nairn and Coverdale, 2005). Working with the media to feature stories that present alternative, stigma-challenging narratives, as well as actively addressing stigmatising media coverage, is an important anti-stigma approach (Ross et al., 2021, 2024a). Social media also presents an important opportunity to tackle stigma towards people with mental illness. Sharing stigma-challenging narratives on these platforms can positively impact attitudes towards people with mental illness (Ross et al., 2019) and allow these stories to reach younger news audiences who are increasingly accessing news on these platforms (Park et al., 2024).

### Impact of news portrayals at a population level

While previous experimental research has examined how the framing and content of individual news stories influence public stigma, the population-level naturalistic exposure to and impact of news reporting on mental health problems remain understudied. Such outcomes and their associations with sociodemographic and lived experience characteristics can only be measured using a large, nationally representative surveys. This includes examining the impact of news stories about mental health problems on people with lived experience, which has mainly been explored in the context of consultation for stigma reduction strategies (e.g. Gronholm et al., 2024; Ross et al., 2024b) where people with lived experience reported viewing the media as a driver of stigma and discrimination. This finding is supported by some experimental studies, e.g., a randomised controlled trial has shown that exposure to a negative news event relating to depression increased stereotype agreement and negative affect in people with depression (Goepfert et al., 2019).

### The current study

This study aimed to determine the population-level prevalence of reported exposure to positive and negative news stories about someone with a mental health problem over a 12-month period, the sources of these portrayals, the impact of being exposed to these news portrayals and how this might vary by sociodemographic variables (age group, gender, education level, language spoken at home and lived experience of a mental illness). Using data collected in the 2025 National Survey of Stigma and Discrimination, this large nationally representative survey is the first to systematically explore these impacts and allows for comparison between experiences reported by population sub-groups, including people with lived experience of mental illness, while minimising selection bias towards people who may have experienced particularly strong negative impacts.

## Methods

### Sample

Data were collected in November 2024 by The Social Research Centre through their Life in Australia participant panel as part of the National Survey of Stigma and Discrimination. The probability-based online panel ensured the survey is nationally representative as panellists were recruited through random digital dialling or address-based sampling. A stratified random sample was drawn from panellists on strata defined by age (18–34, 35–44, 45–54, 55–64, 65+), gender, education (less than a bachelor’s degree, bachelor’s degree or above) and speaking a language other than English at home.

Adults who were aged 18+ and Australian residents were invited to participate via email and SMS phone message. Up to five reminders were sent to panellists during the 2-week study period. Of the 9472 panellists who were invited to participate, a total of 6032 (63.7% completion rate) completed the survey. Participants were who completed the survey were offered a GiftPay voucher or a charitable donation, each to the value of $10 or $20 depending on the number of questions answered and the amount of time this was expected to take, which was based on responses provided due to question branching within the survey. Average survey completion time was 26 minutes.

### Survey questions

The overall survey comprised questions about experiences of mental health problems, attitudes to people with mental health problems and positive and negative experiences connected to mental health problems, with input from SANE’s Lived Experience Advisory Committee. This study focuses on a subset of questions related to news portrayals of mental health problems, with the other findings reported elsewhere (Morgan et al., 2026; Reavley et al., 2026a, 2026b).

*Reported exposure to news stories about people with mental health problems*: All participants were asked two questions to determine their perceptions of exposure to media portrayals of: (1) Over the last 12 months have you heard or seen in the media *positive news stories about a person with a mental health problem?* (positive news story); (2) *Over the last 12* *months have you heard or seen news stories about someone being harmed by a person with a mental health problem?* (negative news story). For the purposes of this study, news stories were categorised as ‘positive’ or ‘negative’ to simplify descriptions of these, which is a common approach used in research examining the impact of media portrayals of mental illness on stigma (Ross et al., 2019). Participants indicated whether or not they had been exposed to a news story about someone with a mental health problem by responding: Yes, No, Don’t know.

*News sources for stories about people with mental health problems*: Participants who reported exposure to a positive or negative news story of a person with a mental health problem were asked about the source for each type of exposure reported. Participants were asked to indicate if they saw or heard the story in each the following traditional and social media news sources. Traditional sources included the newspaper (print or online), a magazine (print or online), radio and TV. Social media sources included Instagram, Facebook, YouTube, TikTok, X (formerly Twitter), WhatsApp, We Chat, Other social media, Online discussion forums or somewhere else (specify). Participants could select all relevant news sources.

*Impact of exposure to news stories about people with mental health problems*: Participants were asked about the perceived impact of each type of news story exposure reported, in order to capture individual experiences of these exposures and examine the sociodemographic and lived experience characteristics associated with impact. This was measured by four questions: (1) *What impact did hearing or seeing positive news stories about a person with a mental health problem in the (a) news (newspaper, magazine, radio or on TV); (b) on social media have on you?; (2) What impact did hearing or seeing news stories about someone being harmed by a person with a mental health problem in the (a) news (newspaper, magazine, radio or on TV); (b) on social media have on you?*

Response options were as follows: (1) a large negative impact, (2) a small negative impact, (3) no impact, (4) a small positive impact, (5) a large positive impact, (6) don’t know. For the purposes of this study, responses were coded into binary impacts. For positive news stories, ‘a small positive impact’ and ‘a large positive impact’ were combined into ‘positive impact’ and all other responses considered ‘non-positive impact’. For negative news stories, ‘a small negative impact’ and ‘a large negative impact’ were combined into ‘negative impact’ and all other responses considered ‘non-negative impact’.

*Lived experience of a mental illness diagnosis*: All participants were asked, ‘Over the last 12 months, have you had any sort of mental health problem?’ Those who responded yes were asked the follow-up question ‘What do you think the problem was?’ and were presented with a list of 27 mental health problems to select from, as well as the option to specify another problem. Those who responded with one or more of the mental health problems in scope of the survey with were then asked: ‘Have you been given a diagnosis from a health professional for this problem?’ Responses were categorised into three categories for the purposes of this study:

1. *No mental illness diagnosis* received from a mental health practitioner or experience of problems considered out of scope for the purposes of this study, one of the aims of which was to compare between people with common versus complex mental illnesses. Problems considered out of scope included experiencing any sort of mental health problem, or who reported experiencing a mental or nervous breakdown, self-esteem or social problems, alcohol and other drug problems, gambling problems or dementia.
2. *Lived experience of a common mental illness diagnosis (common mental illness)*, including depression, anxiety disorders (generalised anxiety disorder, post-traumatic stress disorder, panic disorder, obsessive-compulsive disorder, social phobia), eating disorders, mental illness (not otherwise described), attention deficit and hyperactivity disorder and autism spectrum disorder.
3. *Lived experience of a complex mental illness diagnosis (complex mental illness)* including schizophrenia, schizoaffective disorder, psychosis, personality disorders and bipolar disorder. These conditions are most commonly reported in relation to violence in the news media (Anderson et al., 2018; Hildersley et al., 2020) and are generally more poorly understood and highly stigmatised (Crisp et al., 2005; Switaj et al., 2009).

### Data analysis

The stratified random sample was weighted to ensure that it was representative of the Australian population in terms of the proportions of sociodemographic attributes age, gender, education and speaking a language other than English at home (Australian Bureau of Statistics, 2022). Data obtained via the online surveys were checked for quality (i.e. logic cheques, checking for issues with ‘straight-lining’ and speeding in responses).

Data were analysed using StataSE 18 (StataCorp, 2023) based on weighted data. Descriptive statistics (with 95% confidence intervals) were calculated to describe the sociodemographic attributes of the total sample and the proportions of participants who reported exposure to and impact from news stories about people with mental health problems. Logistic regression analyses were used to analyse if differences in exposure to and impact from news portrayals by sociodemographic characteristics, including gender, age group, education level, language spoken at home and lived experience of a mental illness diagnosis, were statistically significant (*p* < 0.01, with odds ratios and 99% confidence intervals reported to account for multiple comparisons and increase confidence in any effects). Additional logistic regression analyses were used to assess differences in impact across news sources.

## Results

The overall sample comprised 6032 Australian adults aged 18 years or older. Twenty-five percent of the sample had lived experience of a mental illness within the past 12 months, with 23.6% (95% confidence interval [CI] = [22.3, 24.9]) having a common mental illness diagnosis and 2.4% (95% CI = [1.90, 2.90]) having a diagnosis of complex mental illness.

### Reported exposure to news stories about mental health problems

The majority (68.4%, 95% CI = [66.9, 69.8]) of participants reported being exposed to a news story about a person with a mental health problem harming someone else in the past 12 months, while only 33.7% (95% CI = [32.3, 35.2]) reported seeing a positive news story over the same period.

### Sources of news stories about mental health problems

**Figure 1.**
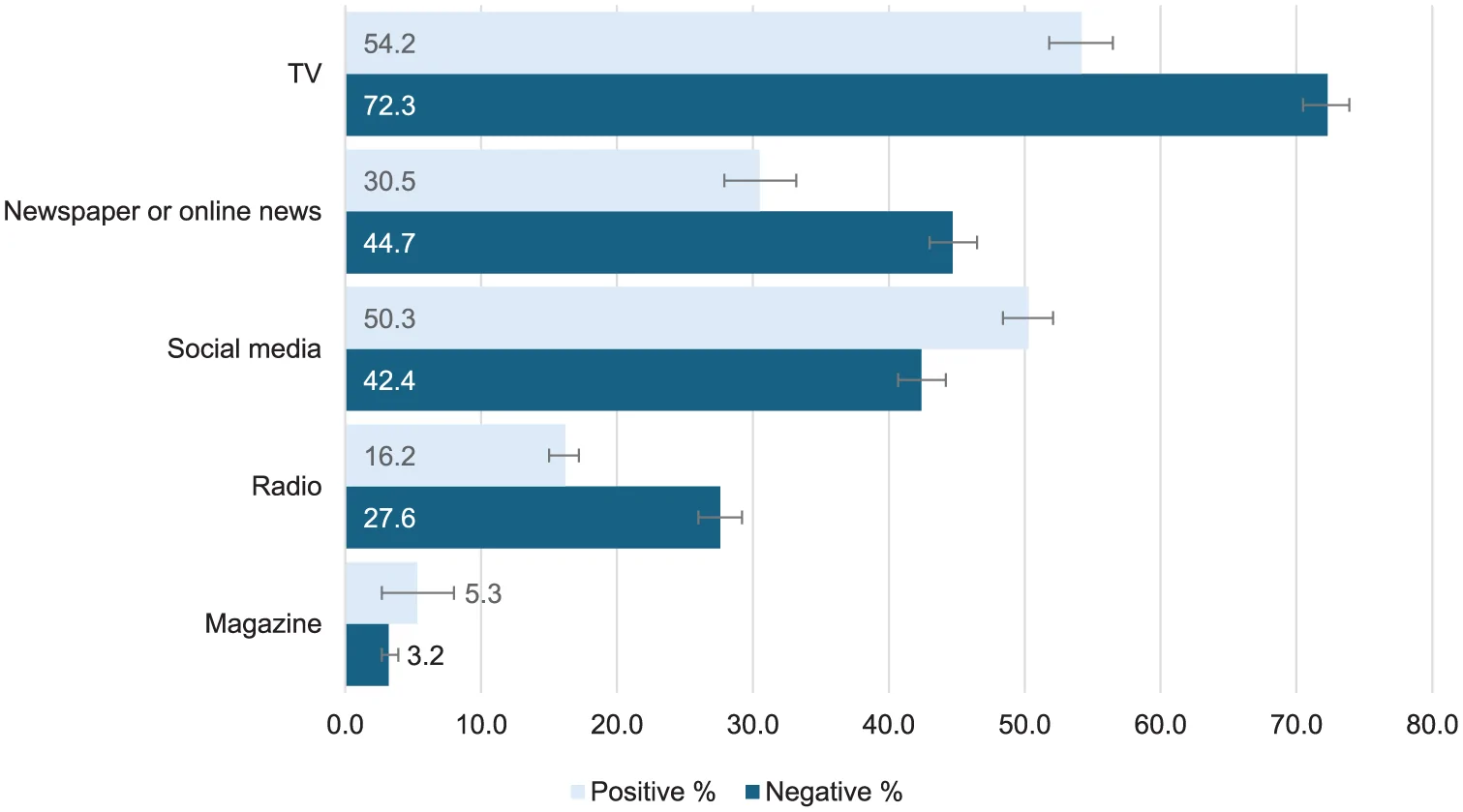
Sources of positive news stories about a person with a mental health problem (*N* = 2055) and news stories about someone being harmed by a person with a mental health problem (negative portrayals, *N* = 4331; 95% confidence intervals).

**Figure 2.**
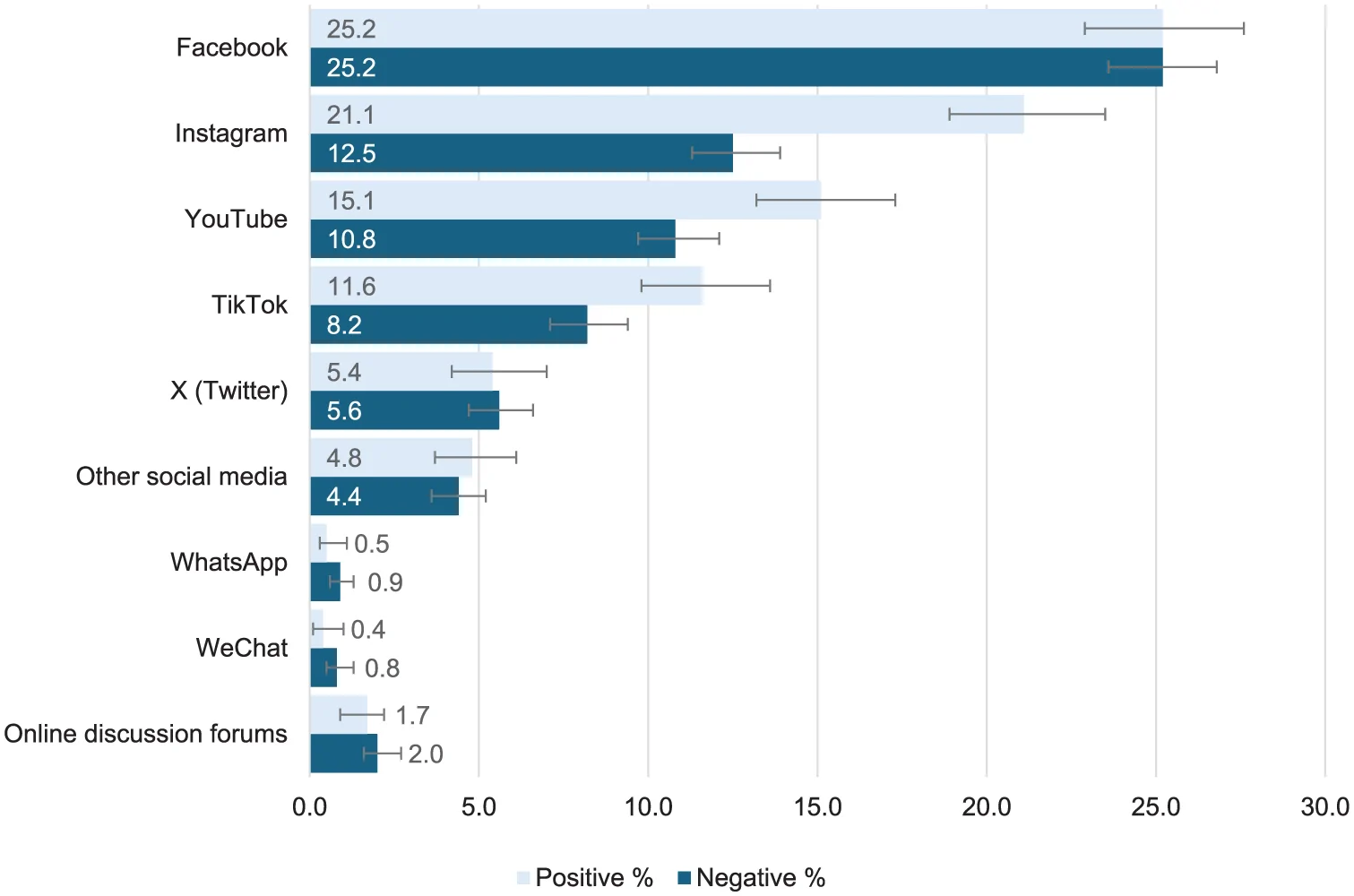
Individual social media sources of positive news stories about a person with a mental health problem (*N* = 921) and news about someone being harmed by a person with a mental health problem (*N* = 1629; 95% confidence intervals).

The main sources of negative and positive news stories about someone with a mental health problem are shown in Figures 1 and 2.

TV was the most commonly reported source of news stories about someone with a mental health problem harming another person (negative news stories, 72%, 95% CI = [70.5, 73.9]), as well as the most common source of positive news stories (54.2%, 95% CI = [51.8, 56.7]). Social media platforms (collectively, 50.3%, 95% CI = [48.5, 52.2]) were reported as the only sources where exposure to positive news stories about people with mental health problems outweighed exposure to negative portrayals (42.4%, 95% CI = [40.7, 44.2]).

Of participants who reported social media as a news source about mental health problems, Facebook and Instagram were the most common sources. Notably, exposure to positive stories outweighed exposure to negative stories on Instagram (21.1%, 95% CI = [18.9, 23.5] vs 12.5%, 95% CI = [11.3, 13.9]), YouTube (15.1%, 95% CI = [13.2, 17.3] vs 10.8%, 95% CI = [9.7, 11.9]) and TikTok (11.6%, 95% CI = [9.8, 13.6] vs 8.2%, 95% CI = [7.1, 9.4]).

### Sociodemographic predictors of reported exposure

Logistic regression analyses were conducted to determine which participants were most likely to report being exposed to positive or negative news stories about someone with a mental health problem, with findings shown in Table 1.

**Table 1.** Reported exposure to positive and negative news stories about someone with a mental health problem (both traditional and social media sources).

		*n* (N = 6029)	Negative news exposure	Positive news exposure	
Predictor variable	Count (weighted)	Odds ratio (99% CI)	*p*-value	Odds ratio (99% CI)	*p*-value
Gender					
Male	2391 (2035)	Reference			
Female	3580 (2915)	0.95 (0.79, 1.15)	0.472	0.88 (0.74, 1.05)	0.062
Other	58 (79)	1.16 (0.47, 2.84)	0.669	1.92 (0.83, 4.47)	0.046
Age group					
18–34 years	1186 (1812)	Reference			
35–64 years	3449 (2903)	1.63 (1.31, 2.02)	**<0.001**	0.86 (0.69, 1.07)	0.070
65+ years	1394 (1314)	4.03 (2.97, 5.47)	**<0.001**	1.00 (0.77, 1.31)	0.963
Education					
Below bachelor	2910 (3962)	Reference			
Bachelor or above	3068 (2016)	1.30 (1.07, 1.59)	**0.001**	1.14 (0.95, 1.37)	0.063
English-speaking background					
English spoken at home	5040 (4859)	Reference			
Language other than English spoken at home	985 (1166)	0.49 (0.39, 0.63)	**<0.001**	0.69 (0.53, 0.90)	**<0.001**
Lived experience of mental illness diagnosis					
None	4486 (4469)	Reference			
Common mental illness	1420 (1421)	0.83 (0.67, 1.04)	0.033	1.31 (1.07, 1.61)	**0.001**
Complex mental illness	126 (142)	0.78 (0.42, 1.45)	0.304	0.86 (0.45, 1.63)	0.538

Bold indicates statistical significance at *p* < 0.01 to account for multiple comparisons.

Reported exposure to negative news stories was significantly higher among people aged 35+ years and with a bachelor’s degree or above; however, these factors did not influence exposure to positive news stories. Exposure to both negative and positive news stories was lower among people from non-English-speaking backgrounds.

People with lived experience of common mental illness were significantly more likely to report exposure to positive news stories and significantly less likely to report exposure to negative news stories than those with no lived experience. Having lived experience of complex mental illness was not associated with exposure to negative or positive news stories about someone with a mental health problem.

### Impact of news stories across traditional news and social media source

Among participants reporting exposure to negative traditional news portrayals, 69% (95% CI = [67.2%, 70.7%]) reported a negative impact. Positive impact from positive traditional news exposures was also reported by the majority of participants but at a slightly higher rate (80.2%, 95% CI = [77.6, 82.5]). Impact from social media news exposures was similar, with impacts again reported by the majority of participants. Sixty-nine percent (95% CI = [66.4, 71.8]) reported a negative impact from negative social media exposures, and 80.8% (95% CI = [77.5, 83.7]) reported a positive impact from positive social media exposures.

The impact of media exposures was examined by news source, with findings outlined in Table 2. Logistic regression analyses revealed that rates of negative impact were similar across all news sources, but was significantly more common from news stories on the radio (odds ratio [OR] = 1.47, 99% CI = [1.16, 1.88]), controlling for other traditional news media sources. Positive impact from exposure to a positive media portrayal was also similar across news sources, with no significant differences in impact found across news sources.

**Table 2.** Proportion of participants reporting impact from traditional news or social media source (weighted %).

Source	Negative impact from negative news source (*n* = 3921) % (95% CI)	*p*-value	Positive impact from positive news source (*n* = 1591) % (95% CI)	*p*-value
Newspaper – print or online	68.9 (66.4, 71.3)	0.674	77.4 (73.2, 81.1)	0.366
Magazine – print or online	67.4 (57.8, 75.7)	0.489	76.6 (65.8, 84.8)	0.597
Radio	74.7 (71.7, 77.5)	**<0.001**	80.5 (74.9, 85.1)	0.910
TV	70.1 (68.2, 72.0)	0.024	81.9 (79.1, 84.4)	0.074
	Negative impact from negative social media source (*n* = 1646) % (95% CI)	*p*-value	Positive impact from positive social media source (*n* = 939) % (95% CI)	*p*-value
Facebook	71.9 (68.3, 75.2)	0.016	83.0 (78.5, 86.7)	0.107
Instagram	71.4 (65.8, 76.3)	0.208	83.8 (78.7, 87.9)	0.120
X (formerly Twitter)	72.2 (64.0, 79.2)	0.373	86.1 (74.9, 92.8)	0.080
TikTok	68.9 (61.7, 75.3)	0.593	85.3 (78.1, 90.4)	0.330
YouTube	70.4 (64.7, 75.5)	0.608	79.7 (73.1, 85.1)	0.889
WhatsApp	92.1 (77.0, 97.6)	**0.010**	86.8 (44.2, 98.2)	0.780
WeChat	92.2 (77.3, 97.7)	**0.005**	37.5 (7.3, 82.2)	0.077
Other social media	66.2 (56.9, 74.5)	0.729	78.9 (66.6, 87.5)	0.824
Online discussion forums	69.7 (55.7, 80.9)	0.633	72.9 (53.5, 86.3)	0.669

Bold indicates statistical significance at *p* < 0.01 to control for multiple comparisons.

With social media sources, negative news exposure WhatsApp (OR = 5.38, 99% CI = [1.01, 28.74]) and WeChat (OR = 6.51, 99% CI = [1.19, 35.73]) were significantly more likely to have a negative impact, controlling for other social media sources. Positive impact from exposure to a positive social media portrayal of someone with a mental health problem was similar across social media sources, with no significant differences in impact found when controlling for other social media sources.

### Sociodemographic predictors of impact from traditional news and social media portrayals of mental health problems

Logistic regression analyses were conducted to determine which participants were most likely to be either negatively or positively impacted by reported exposure to news stories of someone with a mental health problem, with findings shown in Table 3.

**Table 3.** Sociodemographic predictors of participants reporting a negative impact from reported negative news story exposure and positive impact from reported positive news story exposure.

	Impact from traditional media exposure				Impact from social media exposure			
	Negative impact from negative traditional media exposure		Positive impact from positive traditional media exposure		Negative impact from negative social media exposure		Positive impact from positive social media exposure	
Predictor variable	Odds ratio (99% CI)	*p*-value	Odds ratio (99% CI)	*p*-value	Odds ratio (99% CI)	*p*-value	Odds ratio (99% CI)	*p*-value
Gender (Male reference)								
Female	1.55 (1.24, 1.93)	**<0.001**	1.76 (1.16, 2.67)	**<0.001**	1.67 (1.17, 2.39)	**<0.001**	1.02 (0.59, 1.78)	0.914
Other	3.20 (0.84, 12.24)	0.025	1.32 (0.17, 10.36)	0.726	1.89 (0.46, 7.72)	0.245	0.95 (0.20, 4.67)	0.940
Age group (18–34 years reference)								
35–64 years	1.47 (1.07, 2.01)	**0.002**	1.56 (0.88, 2.75)	0.046	1.63 (1.12, 2.39)	**0.001**	1.43 (0.83, 2.48)	0.091
65+ years	1.92 (1.34, 2.75)	**<0.001**	2.95 (1.51, 5.79)	**<0.001**	1.96 (1.10, 3.49)	**0.003**	2.20 (0.76, 6.39)	0.056
Education (Below bachelor reference)								
Bachelor or above	1.02 (0.81, 1.28)	0.856	0.93 (0.61, 1.42)	0.662	0.84 (0.58, 1.20)	0.206	0.99 (0.59, 1.68)	0.955
English-speaking background (English spoken at home reference)								
Language other than English spoken at home	1.31 (0.91, 1.90)	0.056	1.02 (0.53, 1.97)	0.923	1.70 (1.04, 2.78)	**0.006**	0.97 (0.48, 1.96)	0.900
Lived experience of mental illness diagnosis (None reference)								
Common mental illness	0.98 (0.75, 1.30)	0.916	1.45 (0.87, 2.41)	0.061	0.91 (0.61, 1.34)	0.516	0.77 (0.43, 1.36)	0.229
Complex mental illness	1.07 (0.47, 2.47)	0.822	0.67 (0.17, 1.67)	0.451	1.11 (0.39, 3.19)	0.789	0.69 (0.16, 3.00)	0.516

Bold indicates statistical significance at *p* < 0.01 to account for multiple comparisons.

Participants aged 35+ years and identifying as female were more likely to report negative impact from exposure to negative traditional news and social media portrayals, when controlling for other sociodemographic variables. Similarly, participants in these age groups and identifying as female (traditional news sources only) were more likely to report a positive impact from exposure to positive traditional news and social media portrayals. Lived experience of a mental illness diagnosis, education level and English-speaking background were not associated with impact of exposure to news portrayals about people with mental health problems.

## Discussion

Using a nationally representative sample of Australian adults in the 2025 National Survey of Stigma and Discrimination, this study aimed to investigate reported exposure to and impact of news stories about people with mental health problems as well as the sociodemographic and lived experience characteristics associated with these. Findings revealed that the majority of Australians reported being exposed to and impacted by news portrayals of people with mental health problems, with portrayals of a person being harmed by someone with mental health problems (negative stories) being more commonly reported than positive stories.

Most participants (69.0%) reported a negative impact from news stories about someone being harmed by a person with mental health problems and a positive impact from positive news stories about mental health problems (80.2%). This finding is largely consistent with impacts from exposure to news stories about people with mental health problems in past research on public attitudes. Negative news portrayals have been broadly found to negatively impact public attitudes about people with mental health problems through increased stigma and increased desire for social distance, and positive news portrayals have been found to have positive impacts on stigma and public attitudes (Corrigan et al., 2013; Dietrich et al., 2006; Gwarjanski and Parrott, 2017; McGinty et al., 2018; Morgan and Jorm, 2009; Ross et al., 2019). These findings further demonstrate that news stories about mental health problems affect the majority of news audiences, not just those with lived experience of mental illness. This finding therefore emphasises the importance of ensuring media portrayals of people with mental health problems are accurate and responsible and the importance of featuring positive portrayals in mainstream news to promote positive impacts and mitigate negative impacts on stigma.

Participants aged 35+ years and identifying as female were found to be more likely than those aged 18–34 and males to report being impacted by both positive and negative media portrayals of people with mental health problems. This finding may perhaps reflect differences in news consumption, with younger people more likely to engage with news on social media, where findings showed positive news stories about mental health problems are more prevalent, while older age groups prefer more traditional news sources (Park et al., 2024) which were associated with more negative impacts. Lived experience of a mental illness diagnosis was not associated with impact, but instead findings showed that news audiences broadly reported being impacted by exposure to news. News organisations therefore need to consider audience-wide impacts when developing news stories about people with mental health problems, being aware that positive portrayals have a broad positive impact while portrayals of violent acts need to follow responsible reporting guidelines (Everymind, 2020; Ross et al., 2020) to mitigate negative impacts on public attitudes and behaviours.

The majority of participants (68%) reported exposure to a news story about someone being harmed by a person with a mental health problem. Exposure to negative portrayals far outweighed exposure to positive stories (34%). This finding arguably suggests that exposure to portrayals of someone harmed by a person with a mental health problem is more widespread, and these portrayals potentially have a stronger impact on memory and recall, and perhaps even a larger influence on stigma (e.g. Morgan and Jorm, 2009).

Social media was the only news source where exposure to positive stories outweighed exposure to negative stories. This finding suggests that negative portrayals continue to dominate the mainstream news through more traditional news sources, including news websites and newspapers, TV and radio. Specifically, positive portrayals of people with mental health problems were most commonly reported on Instagram, YouTube and TikTok, which are video-based social media platforms. The video format may facilitate more feature-style portrayals about lived experiences of people with mental health problems (e.g. Tudehope et al., 2024) compared to other text-based social media platforms (Facebook, e.g. Lyons et al., 2025). This further contrasts to traditional news sources where a negativity bias influences the production and consumption of news (Andersen et al., 2024; Robertson et al., 2023). Furthermore, personalisation of newsfeeds on social media platforms due to algorithms that aim to maximise engagement may also influence more positive news exposure and consumption broadly (Wilding et al., 2018).

In interpreting these findings, it is important to consider the Australian media context for the survey period. In April 2024, 7 months prior to the survey being conducted, violent attacks occurred at a Sydney shopping centre where the perpetrator was reported by police to have a history of ‘mental health issues’. This was later confirmed by their family as having a ‘history of schizophrenia’, with declining mental health in recent years since ceasing treatment. While the media coverage was found to mostly align with responsible reporting practices (Wake and Ross, 2025), the incident received prominent news coverage which was ongoing in the wake of a public inquest into the tragic event. Such extensive media coverage would likely have influenced exposure to these negative portrayals within the survey period than in a typical year, where such a rare event typically does not occur nor attract such immense media coverage.

To highlight strengths, this study was the first nationally representative study to investigate exposure to and the impact of media portrayals of mental illness on people with and without lived experience of a diagnosed mental illness. This study has revealed that negative media portrayals of mental illness are prominent and the negative impact these have at a population-level. Further research is needed into how participants were impacted by exposure to these positive and negative news portrayals, and at what levels of exposure (‘dose’, or amount of news stories seen/heard) influenced such impacts. The measure of exposure was likely subject to self-report biases, including recall and negativity biases, whereby participants may overestimate their actual exposure to negative news stories and stories of most interest to them (e.g. Wirz and Blassnig, 2025). Using this measure, participants provided an approximate (rather than exact) response regarding their exposure to relevant news stories over the past 12 months. Given the aim of this study was to capture data on reported exposure and impact in a real-world setting, using brief measures provides a meaningful approximation to allow examination at a population level and exploration of sociodemographic and lived experience characteristics associated with reported impact. Furthermore, the measure of impact did not distinguish the type of negative impact experienced, such as distress about the incident itself, fear towards people with mental illness, impact on individual mood, impact on self-stigma or distress about being further stigmatised, which is a key limitation of the study. Future research should examine how negative and positive impacts were interpreted among participants.

While no differences in impact from news exposure across lived experience groups were found in this study, it is possible that this lack of meaningful difference was due to a limited number of participants in this group (*n* = 126 reported lived experience of complex mental illness, with 76 reporting relevant news exposure) and therefore lack of statistical power. Given that people with complex mental illness are more commonly portrayed in relation to violence and crime than people with common mental health problems, it was expected that these portrayals would have a stronger negative impact on people with lived experience of these diagnoses. Future research should systematically explore impacts of news exposure among people with lived experience of complex mental illness, particularly on self-stigma, to better understand any direct impacts from these. While outside of the scope of this study, future research should also examine exposure and impact related to media coverage of substance use and gambling problems, as these are also highly stigmatised and warrant further investigation.

Findings reveal that exposure to news stories about someone with mental illness harming someone else is highly memorable and likely to increase stigma. It is therefore important that media guidelines for responsibly reporting on mental illness and crime are fully implemented to avoid perpetuating harmful stereotypes about people with mental illness. This should include education for journalism students and media professionals, as well as allowing regulatory bodies to govern media portrayals, as has been achieved through the Australian Press Council standards for responsible reporting on suicide (Australian Press Council, 2021). More positively framed news stories are also needed to improve awareness and understanding of mental illness, and promote stigma-challenging narratives. Media professionals should be encouraged and supported in developing such stories, with training and guidance provided to both media professionals and people with lived experience in advocacy roles to ensure their media engagement is a positive and safe experience (Everymind, 2025; Ross et al., 2024b).

## Conclusion

This study demonstrates that reported exposure to negative news portrayals remains dominant among Australians, and these stories negatively impact news audiences regardless of lived experience of mental illness. Exposure to positive news stories is far less common, but these have an important and necessary role in educating and increasing understanding of mental health problems. Given the negative impact of negative news stories on news audiences broadly, these need to follow responsible reporting guidelines to ensure they are accurate and aim to reduce negative impacts. A renewed focus on generating and promoting positive and stigma-challenging news stories is needed to increase exposure to and subsequent positive impacts on attitudes and understanding of mental health problems.
